# Multidisciplinary Care for Acute Kidney Injury Survivors Following Hospital Discharge

**DOI:** 10.1016/j.xkme.2023.100619

**Published:** 2023-03-02

**Authors:** Joseph Lunyera

**Affiliations:** Division of General Internal Medicine, Department of Medicine, Duke University School of Medicine, Durham, NC

Related article, p ●

Survivors of acute kidney injury (AKI) experience an increased risk of subsequent adverse outcomes, including the incidence of chronic kidney disease, cardiovascular disease, and death.^1^ There are no evidence-based clinical practice guidelines specifically addressing clinical care for AKI survivors following hospital discharge.^2^ For this reason, clinical care pathways for AKI survivors remain undefined, resulting in varying clinical care practices among providers. These variations in practice include the timing of follow-up visits to clinics, nephrology referral, laboratory monitoring of kidney function, and patient education after discharge.^3^ However, formal investigations of care for and the experience of AKI survivors after discharge are beginning to emerge,^4^ with the potential to generate an evidence base that can define clinical care pathways for AKI survivors following hospital discharge. Ultimately, defined and standardized clinical care pathways for AKI survivors after discharge could optimize clinical outcomes in this patient population with a high number of unmet medical needs.^5^

An important addition to the growing body of evidence on clinical care for AKI survivors has been described in this issue of *Kidney Medicine*. In their article, May et al^6^ reported the findings of a mixed-method study that sought to identify nephrologists’ and primary care providers’ perspectives on best practices for AKI survivor care following hospital discharge. The quantitative component of the study compared knowledge and practices regarding AKI survivor care between nephrologists and primary care providers based on their responses to survey questions about 3 hypothetical clinical vignettes of AKI survivors. The survey questions assessed the participants’ knowledge and practices in the following 3 domains of care: timing of follow-up laboratory monitoring of kidney function (ie, within 14 days, 1-2 months, 6 months, or a later timing), medication and comorbidity management, and nephrology referral at the time of discharge. The qualitative component of this mixed-method study elicited nephrologists’ and primary care providers’ recommendations on strategies for optimizing collaborative AKI survivor care using an open-ended interview guide. Of note, these open-ended interviews were specifically designed to expand upon findings from the above-mentioned quantitative survey by highlighting the best practices for collaborative AKI survivor care in a multidisciplinary team setting.

There were several important findings from this study, which had 148 survey respondents (24 nephrologists, 105 primary care providers, and 19 unknown) and 17 qualitative interviewees. First, the primary care providers, compared with the nephrologists, less often recommended nephrology referral for follow-up care of AKI survivors with proteinuria at discharge. For example, only 30% of the primary care providers versus 63% of the nephrologists recommended nephrology referral for a hypothetical 65-year-old patient with stage 2 AKI that was partially resolved and proteinuria at discharge. In contrast, most primary care providers (90%) and all the nephrologists recommended nephrology referral at discharge for a similar hypothetical case but with comorbid conditions in addition to proteinuria at discharge. These data suggest that a substantial proportion of primary care providers and some nephrologists do not recognize the prognostic importance of proteinuria in the risk of progressive loss of kidney function following an AKI episode.^7^ Of note, this prognostic role of proteinuria in the progression of chronic kidney disease is independent of known risk factors for chronic kidney disease such as baseline estimated glomerular filtration rate and other comorbid conditions.^7^ Indeed, several studies have documented a low rate of albuminuria or proteinuria testing among AKI survivors, ranging from 6% after discharge in 1 study^8^ to 17% in another study.^9^ Thus, there is a need to increase awareness (eg, through educational initiatives) among providers, particularly those outside of the nephrology community, on the importance of albuminuria or proteinuria as a tool for identifying patients with a high risk of kidney disease progression and who would benefit from prompt nephrology referral.^10^

Another important finding from this study was that not all the providers who participated in the survey (only 63% of the nephrologists and 83% of the primary care providers) recommended follow-up laboratory monitoring of kidney function within 14 days after discharge for a hypothetical 40-year-old patient with AKI who required dialysis and was not dependent on dialysis at discharge. For reference, an expert panel from the 22nd Acute Disease Quality Initiative recommended that laboratory monitoring of kidney function be performed within 7 days of hospital discharge in patients with AKI requiring dialysis, regardless of dialysis dependency at discharge.^5^ However, the open-ended interviews identified important barriers to care that might explain why some providers are dissuaded from recommending shorter timelines for monitoring of kidney function; these include insurance coverage issues (out-of-network, high-deductible plans), problems with physical access to laboratories (traveling long distance, difficulty in taking time off work), and problems with timely availability of test results. Thus, effective interventions that address these roadblocks may need to be prioritized in future efforts to define optimal follow-up care of AKI survivors after discharge.

Last but perhaps one of the most important findings that emerged from the interviews was the providers’ recommendation for collaborative care for AKI survivors. It has been suggested that a multidisciplinary approach to postdischarge care of AKI survivors yields the best clinical outcomes for these patients.^5^ This proposed multidisciplinary approach involves a collaborative partnership among providers in the fields of nephrology, primary care, pharmacy, nutrition, and social work. However, few studies have formally investigated the best practices for integrating contributions from this multidisciplinary team of providers to the care of AKI survivors. In their analysis of themes that emerged from the open-ended interviews, May et al^6^ identified the need for integrating input from a multidisciplinary team as an important priority among both nephrologists and primary care providers. For example, 1 nephrologist emphasized the need to engage pharmacists in the review of medications for AKI survivors, particularly medication reconciliation for individuals on multiple medications. Other providers emphasized the importance of involving dietitians to support patient education initiatives. These insights on the role and importance of a multidisciplinary team in the care for AKI survivors after discharge will be essential moving forward for the development of care pathways that put patients at the center of their care. Furthermore, it should be noted that a multidisciplinary approach to AKI survivor care could lessen the need for referral of patients with a low risk of kidney disease progression to nephrology, which has been deemed unnecessarily burdensome for health systems (given the shortage in the nephrology workforce)^11^ and some patients (eg, long travel time to referral centers and patients’ reluctance to add specialists to their care team).^5^
